# Predictors of concussion reporting intentions in adolescent hockey players

**DOI:** 10.1007/s12144-023-04316-7

**Published:** 2023-02-11

**Authors:** Natalie D. Macdonald, Oliver Baumann

**Affiliations:** School of Psychology, Bond University, Gold Coast, QLD Australia

**Keywords:** Sport Injury, Athletes, Attitudes, Knowledge, Gender

## Abstract

Young athletes who do not report a concussion injury are at greater risk for a prolonged recovery time and further neurocognitive impairments. Despite the seriousness of the issue and the scale of the problem, not enough is known about the behavioural underpinnings of concussion underreporting in minor athletes. This paper aims to apply the Knowledge, Attitude, and Behaviour (KAB) framework to the issue of injury reporting in adolescents, with the specific purpose of exploring to which degree concussion knowledge, concussion attitudes, and gender affect concussion reporting intentions of both male and female athletes. We recruited 97 young athletes between the ages of 14 and 19 (M = 16.22, SD = 11.06) from the Okanagan Hockey Academy (Canada) and employed a self-administered supervised survey approach to measuring the target variables. A hierarchical multiple regression was conducted, and consistent with the prior literature, females were more likely to report a sport-related concussion than males. It was further found that attitudes around concussions (i.e., taking concussions seriously) were significant predictors of concussion reporting intention. At the same time, there was no significant relationship between concussion knowledge and concussion reporting intention. These results highlight that knowledge about concussion symptoms is insufficient to warrant proper injury reporting. It will therefore be essential to work on changing the attitudes of young athletes regarding the significance of concussions to achieve meaningful behavioural change.

A concussion is defined as a traumatic brain injury induced by biomechanical forces and is considered the mildest form of traumatic brain injury (McCrory et al., 2017). The term ‘mild’ does not reflect how severe the injury is but rather the lack of structural brain damage. A sports-related concussion may be caused by a direct impact to the head, face or neck, or even to other parts of the body, and commonly results in the rapid onset of short-lived neurological impairment and symptoms (Halstead et al., 2018).

The multivariable nature of a sport-related concussion is not necessarily associated with a loss of consciousness but rather with various physical, cognitive and emotional symptoms, such as headaches, vision disturbances, memory and concentration difficulties, changes in behaviour and alterations in sleep patterns (Lazzarino et al., 2012). The Berlin consensus statement has found multiple indicators of concussion that have been observed and documented. These indicators include disorientation and confusion that occurs instantly after the concussive event, impaired balance up to one day after a concussive event, slower reaction times and/or impaired memory and verbal learning up to two days after a concussion injury (McCrory et al., 2017). Neurological symptoms that occur post-concussion are believed to be a result of a functional microstructural injury of the axon and unrelated to macroscopic neural damage (Halstead et al., 2018). There are no biomarkers or scans, however, that are currently verified or validated that can confirm a concussion injury, meaning they are undetectable in bloodwork or on neural imaging scans (e.g. MRI and CT scans). It is important to note that an absence of symptoms does not mean an absence of injury, which is what many athletes assume before returning to their sport. If not fully healed from a concussion injury, athletes put themselves at risk of further and more serious injury. After a concussion injury, healing is still required even after the alleviation of symptoms. It has been found that symptoms tend to be absent at seven days post-injury; however, metabolic recovery in the brain does not occur until 22 to 30 days post-injury (Vagnozzi et al., 2010).

Younger athletes are at greater risk of concussion since research has shown that the developing brain is more susceptible to injury than the adult brain. For instance, studies conducted on rats have shown an increase in cognitive impairment and injury in the axons of young rats compared with mature rats (Prins et al., 2010). It has also been suggested that during brain development there is an ongoing maturational change in myelination, which insinuates that the immature brain is more vulnerable to axonal injury (Halstead et al., 2018), while weaker neck muscles in paediatric populations may impair the ability to stiffen the neck to brace for impact, further increasing risk of concussion (Collins et al., 2014).

It is possible to heal completely from a concussion with no neurological deficits; however, if a concussion injury goes unreported or undiagnosed, it can carry a risk of additional, more complicated and longer-lasting effects on the brain, such as mental health changes and neurological deficits (Terwilliger et al., 2016). More specifically, research has suggested that continued participation in a sport while still experiencing symptoms can lead to prolonged recovery. In contrast, a second concussion during the first recovery can have magnified effects on the brain (Kroshus et al., 2014). Research has also shown that younger athletes and adolescents need longer recovery after a concussion than college and adult athletes (Halstead et al., 2018). Concerningly, the same study has shown that 10–38% of younger athletes returned to play on the same day of their injury. In addition, it has been suggested that roughly 50% of concussions go unreported (Register-Mihalik et al., 2013). Despite the seriousness of the issue and the scale of the problem, little is known about the behavioural underpinnings of concussion underreporting in minor athletes. Firstly, reporting behaviour could be underpinned by *concussion knowledge*, i.e. an athlete must be able to identify the signs and symptoms of a concussion to report it. Secondly, reporting behaviour could be underpinned by *concussion attitudes*, i.e. an athlete must take the issue of concussion seriously enough to act. Whereas several studies have looked at the effects of interventions on concussion knowledge, attitudes, and reporting behaviour (for an overview see Beran & Scafide, 2022), there needs to be more research on the differential effects of concussion knowledge and concussion attitudes on reporting behaviour. However, understanding the relative impact of knowledge vs. attitudes on reporting behaviour in young athletes is crucial for understanding reporting behaviours and developing effective interventions. Carpenter and colleagues (2020) conducted a cross-sectional regression analysis to investigate this question and found no effect of either concussion knowledge or attitudes on reporting behaviour. Given that the sample in this study consisted of only 40 (all male) participants, the analysis might have lacked sensitivity. The current study’s rationale was e in a larger (mixed-gender) sample.

Moreover, we employed the Knowledge, attitude and Behaviour framework (KAB) as the theoretical foundation for our investigation. The KAB is a well-established theoretical model in the field of health education, used as a conceptual framework to predict behaviour based on measurements of knowledge and attitude (Bettinghaus, 1986). It posits that knowledge is the essential foundation for health-related behaviour. However, more than knowledge is needed to lead to behavioural change. I.e. in addition to knowledge, individuals must believe in the personal threat of illness and the effectiveness of the health behaviour recommendations for beneficial behaviours to be adopted (Janz & Becker, 1984). Therefore, our study aimed to apply this model to explore to which degree concussion knowledge and attitudes affect concussion reporting intentions of male and female athletes.

## Method

### Participants

The sample was made up of both male and female hockey players from the Okanagan Hockey Academy. 64 Males (66%) and 33 Females (34%) voluntarily participated between the ages of 14 and 19 (M = 16.22, SD = 11.06). According to a G-power 3.1 (Faul et al., 2009) analysis, a sample of 85 participants would be sufficient to detect a medium-sized effect with 80% statistical power (regression model with 4 predictors). The majority of the participants were 17 years of age (40.2%), followed by 16 years (28.9%), 15 years (17.5%), 14 years (7.2%), 18 years (5.2%) and 19 years (1%). No other demographic variables were collected for the purpose of this study. In Canada, players under the age of 16 years are required to have parental written consent to participate in a study. Therefore, parental consent forms were required to adhere to the ethical principle of protecting vulnerable individuals (Field & Behrman, 2004). The players under the age of 16 years who obtained parental consent also needed to sign their consent form to signify their autonomy and their right to give assent to participate (Field & Behrman, 2004). Participants aged over 16 years only had to sign their consent forms to participate in the research study.

### Materials & design

The survey was administered in person using paper copies, thus increasing the quality of the data collection. The survey consisted of 43 items, statements divided into four predictor variables, i.e. Age (years), Gender (M/F/O), Concussion Attitudes (9 items), and Concussion Knowledge (25 items), and the criterion variable Concussion and Injury Reporting Intention (7 items).

Concussion knowledge was measured using True/False statements. Correct answers counted 1 point each, so scores on this scale ranged from 0 to 25, with higher scores indicating greater knowledge. In contrast, Concussion Attitudes and Injury Reporting Intention are measured using statements in the form of a five-point Likert scale ranging from 1 (strongly disagree) to 5 (strongly agree). Higher scores on Concussion Attitudes indicate that respondents take the issue of concussion more seriously. In comparison, higher average scores on Reporting Intentions indicate that the respondents are more likely/willing to report sporting injuries. The internal consistency of the two Likert scales was very good, i.e. the Concussion Attitudes scale had a Cronbach’s alpha of 0.886, and the Reporting Intentions scale had a Cronbach’s alpha of 0.844. The Concussion Knowledge was a performance scale, so Cronbach’s alpha was not applicable here. Given that the constructs of interest (i.e. knowledge, attitudes, and intentions) were all considered changeable, no re-test reliability measures were obtained.

### Procedure

The study was given ethical approval on 19 January 2021 by the Institution’s Human Research Ethics Committee (approval number OB00033). The study employed an exhaustive convenience sampling approach, i.e. we aimed to measure the entire cohort of adolescent hockey students at a hockey training institution. The study was conducted in a private room at the Okanagan Hockey Academy training facility. All potential participants under the age of 16 years were required to hand in a parental consent form, or they could not participate. Those who were able to participate were given a consent form and an explanatory statement to consent to the data being used. The researcher went through the explanatory statement with all participants to ensure they understood the inclusion and exclusion criteria, the task, the expected benefits and potential risks and the information about confidentiality.

After signing the consent form, the participants were given a paper copy of the survey to complete. They were asked not to discuss their answers with other participants and not to speak about the survey with other athletes on different teams until after the data collection part of the study was complete. Each session took around 30 min, including an explanation of the survey, signing the consent forms, and completing the survey. Due to COVID-19 restrictions, all participants, coaches, managers and the researcher were required to wear a mask during the data collection period. The researcher also ensured that all pens, chairs and work surfaces were cleaned between sessions to ensure the safety of the participants.

### Data analysis

A hierarchical multiple regression including four blocks was run on the data. Step one included Age, step two added Gender, step three added Concussion Knowledge, and step four added Concussion Attitudes. The Mahalanobis distance and Cook’s distance were calculated to test univariate and multivariate normality.

## Results

Screening of the data indicated a 100% completion rate, i.e. no missing data. Further, all four continuous variables were screened for severe outliers (criterion 3x interquartile range), however, none were detected. The maximum Mahalanobis distance was 11.10, well below the critical value of 18.47 (df = 4), and the maximum Cook’s distance was 0.07, indicating that there were also no multivariate outliers. Bootstrapping of 95% confidence intervals (1,000 samples) showed no change to the regular *B* coefficients, therefore suggesting no issues regarding normality. Tolerance values of ≥ 0.879 indicated no concerns regarding multicollinearity. Please see Table 1 for a correlation matrix of continuous predictors and outcome variables, showing significant positive relationships between age and concussion attitude, suggesting that participants took the issue of concussion more seriously with increasing age. In addition, there was a positive relationship between concussion attitudes and concussion reporting, suggesting that if participants took the issue of concussion more seriously, they were also more likely to report injuries.

**Table 1:** Correlation matrix of continuous predictors and outcome variables

	Age	C_KNOW	C_ATT	C_REPORT
Age	-			
C_KNOW	− 0.052	-		
C_ATT	0.206*	− 0.166	-	
C_REPORT	0.0133	0.043	0.557***	-

*Note.* * *p* < .05, ** *p* < .01, *** *p* < .001

In the first regression block, age accounted for 1.8% of the variance and did not significantly predict reporting intention (Δ*R*^2^ = 0.018, Δ*F* (1,95) = 1.718, *p* = .193). Gender was entered in step two. This was added separately to age to reveal if either age or gender had a significant effect on the reporting of injury and accounted for an additional al 7.2% of the variance, significantly predicting reporting intention (ΔR^2^ = 0.072, ΔF (1,94) = 7.470, *p* = .007). In step 3, concussion knowledge accounted only for a nonsignificant increase of reporting intention (ΔR^2^ = 0.001, ΔF (1,93) = 0.051, p = .821). Finally, in step 4, concussion attitudes accounted for an additional 25.7% of the variance and significantly predicted reporting intention (ΔR^2^ = 0.257, ΔF (1,92) = 8.747, p < .001). In combination, the four predictor variables explained 34.8% of the total variance (R^2^ = 0.348, adjusted R^2^ = 0.320, ΔF (4,92) = 12.271, p < .001). The standardised regression equation of the final model is y = 0.029*Age + 0.139*Gender + 0.120*C_KNOW + 0.541*C_ATT+-0.872. Please see Table 2 for the unstandardised (B) and standardised (β) regression coefficients and the squared semi-partial (or ‘part’) correlation (sr^2^) for each predictor in the regression model.

**Table 2:** Coefficients and squared semipartial correlations

		*B*	*β*	*t-value*	*p*-value	sr^2^
1	Age	0.072	0.133	1.311	0.193	0.018
2	Age	0.073	0.134	1.363	0.176	0.018
	Gender	0.325	0.269	2.733	0.007	0.072
3	Age	0.073	0.135	1.366	0.175	0.018
	Gender	0.322	0.267	2.682	0.009	0.070
	C_KNOW	0.005	0.023	0.227	0.341	< 0.001
4	Age	0.016	0.029	0.333	0.740	< 0.001
	Gender	0.169	0.139	1.599	0.113	0.018
	C_KNOW	0.026	0.120	1.391	0.167	0.014
	C_ATT	0.700	0.541	-6.025	< 0.001	0.2547

## Discussion

Despite the considerable health risks of concussion and the scale of the problem, not enough is known about the behavioural underpinnings of why young athletes underreport concussions. More specifically, it is unknown how much concussion knowledge and attitudes towards concussion determine reporting intentions. Therefore, our study aimed to apply the KAB framework (Bettinghaus, 1986) to predict injury-reporting behaviour in young athletes based on concussion knowledge and attitudes. Whereas a previous study (Carpenter et al., 2020) had approached this question and did now identify any relationship between knowledge and attitudes related to concussion since it might have been due to the relatively small sample size. Furthermore, the study by Carpenter and colleagues included an all-male sample, limiting the generalisability of the findings. Our study, therefore, provides new data on this critical issue.

Firstly, in terms of demographic variables, our findings were in line with previous research, indicating that females were more likely to report a concussion than males (Beidler et al., 2018; Brown et al., 2015; Halstead et al., 2018). This sex difference is likely explained by the cultural differences between male and female athletes, causing a reluctance in male athletes to report a concussion (Wallace et al., 2017a). Nevertheless, it is essential to note that despite these findings, future studies should not just be focused on males. Female athletes are an indispensable part of concussion research, and concussion education should be aimed at male and female attitudes.

Our study’s most important and novel finding was that attitude significantly impacted concussion and injury reporting intention in young athletes, while concussion-related knowledge did not. This indicates that a high level of concussion knowledge does not necessarily mean a high likelihood of reporting a concussion. Similarly, (Mrazik et al., 2015) found that athletes would present with high concussion knowledge, with 95% of those athletes indicating that they should stop and tell someone. However, the study found that only 43% of athletes followed the correct protocol when reporting a sports-related concussion (Mrazik et al., 2015). These findings illustrate that even though an athlete may present with good knowledge and a working understanding of what a concussion is, it does not change what they may do. A key suggestion of our results is that if the athletes possessed a different attitude towards concussions, their intentions to report a concussion would increase.

Attitudes regarding minimalising concussion risks could be, to some degree, a cultural artifact. A qualitative study of Canadian minor hockey players found that under-appreciation of injury-related health risks was prevalent among minor hockey players and coaches. Exemplified by a coach’s saying, ”you don’t know whether it’s a concussion or just a headache or something” (Cusimano et al., 2017). If a coach, mentor, or role model (e.g., a parent, teacher or friend) of an athlete has a ‘man up’ or ‘rub some dirt in it’ attitude, then the athlete is more likely to develop a similar attitude towards reporting any injury, including concussions. Wallace and colleagues (2017b) also found that athletes did not want to report a concussion injury mainly because they didn’t want their coach to get mad or to think they were weak, and they did not want to upset their parents.

It is important to highlight that our findings were obtained in sample of hockey players, however, different team sports, such as football, rugby, lacrosse and soccer, may have varying attitudes towards concussions and may impact concussion reporting intentions. Depending on the sport, there may be different attitudes towards concussion and differences in the education around concussion, which should be investigated by future studies. Research has found that American football has the highest number of traumatic brain injuries out of all the sports played in the United States. At the same time, hockey and rugby also have an increased risk of concussion injuries (Daneshvar et al., 2011). Individual sports may also show different levels of concussion reporting intention as the athletes may feel less social pressure due to a lack of teammates. However, they may feel more internal pressure as they rely on themselves for athletic success. Also, their coach or parents may pressure them to return to play sooner than they should.

The attitude and knowledge of educational, medical, and administrative staff regarding concussions are also likely to be determining factors in the reporting intention of athletes. Therefore, increasing knowledge and changing attitudes from front-line workers to the highest levels of administration could be vital in changing the overall culture and increasing concussion reporting. Organisational cultural change is not only underpinned by what the members have learned but also by what they believe (Schein, 2010). Only shared patterns of belief will make it possible to change perceptions and behavioural patterns that ultimately lead to better outcomes (Davis, 1984; Hofstede et al., 1990); i.e. in this case, the reduction of the severity and long-term consequences of concussions).

In addition, it might be a fruitful strategy to educate parents. When looking at the knowledge of parents around concussions, one study found that even though 94% of the parents surveyed reported that their child never had a concussion, only 13% were able to correctly identify all statements regarding concussions (Mannings et al., 2014). Interestingly, two-thirds of these parents did not realise that a concussion is considered a mild traumatic brain injury, and 42% were unaware that a concussion could be achieved by something other than a direct hit to the head (Mannings et al., 2014). Despite being given high-quality instructions after being discharged from a medical facility, parents were still unclear about the follow-up instructions (Thomas et al., 2018). Many parents could not identify common concussion symptoms and misidentified serious symptoms. Educating parents on the signs and symptoms of a concussion could help with increased reporting and the quality of an athlete’s recovery from a concussion injury.

It is important to note that while our study focussed on the role of knowledge and attitudes (in addition to gender), previous research has identified other potential facilitators and barriers to sports injury reporting. For instance, it has been shown that the availability of athletic trainers is a significant facilitator of injury reporting (McGuine et al., 2018). In addition to education, it will be essential to ensure a good supervision ratio for young athletes. Another psychological barrier to injury reporting in high school students, identified in a systematic review (Clark & Stanfill, 2019), is the fear of being excluded from playing and letting one own team down. These issues should therefore be part of future education aimed at changing attitudes regarding the seriousness of concussions compared to the perceived risks of reporting injuries.

In summary, the critical novel finding of our study is that, at least in young athletes, knowledge about concussion symptoms is insufficient to warrant proper injury reporting. These new insights are underpinned by several strengths of our study, including the in-person survey approach, which led to high quality data (i.e. 100% completion rate), and a sufficiently powered mixed-gender sample (> 80% power to detect a medium effect). Our study’s key implication is that it will be essential to work on changing young athletes’ attitudes regarding the significance of concussions to achieve meaningful behavioural change. Moreover, changing educational, medical, and administrative staff attitudes regarding concussions could be vital in changing the overall culture and increasing concussion reporting. Organisational cultural change is not only underpinned by what the members have learned but also by what they believe (Davis, 1984; Hofstede et al., 1990; Schein, 2010). Only shared beliefs will make it possible to change perceptions regarding the severity and long-term consequences of concussions. Finally, previous health research has highlighted that changing attitudes is only the first stage for changes in health behaviour. According to the Health Action Process Approach (HAPA) model, education that changes attitudes leads to intentions to change behaviour (i.e. the motivational phase). But for actual change behaviour to occur, further education needs to happen that trains the planned and effective enactment (i.e. the volitional phase) of appropriate behavioural responses (Schwarzer, 1992, 2008a). I.e. players, parents, and coaches will need to practice pragmatic behavioural routines that include simulations of real-life challenges and resistance to injury reporting (Schwarzer, 2008b).

Determining the most effective avenue for delivering concussion education will also be crucial to increase the reporting of concussions. A multimodal delivery that presents the information in different ways, such as through online videos, in-person education sessions and discussions around concussions, may be beneficial (Moro et al., 2019). Further, including a concussion education programme in schools as part of the physical education curriculum could also help educate non-athletes (Schall, 1994). The design and evaluation of adequate education and training programs that address motivational and volitional aspects of behavioural change warrant further research and funding. However, this is well justified, given the importance of reducing long-term concussion-related brain damage.

## Data Availability

The data is made available via Open Science Framework (DOI 10.17605/OSF.IO/BF6ZG). The questionnaire is available from the corresponding author upon request.
